# Interleukin 4 Controls the Pro-Tumoral Role of Macrophages in Mammary Cancer Pulmonary Metastasis in Mice

**DOI:** 10.3390/cancers14174336

**Published:** 2022-09-05

**Authors:** Carolina Rodriguez-Tirado, David Entenberg, Jiufeng Li, Bin-Zhi Qian, John S. Condeelis, Jeffrey W. Pollard

**Affiliations:** 1 Department of Developmental and Molecular Biology, Albert Einstein College of Medicine/Montefiore Medical Center, Bronx, NY 10461, USA; 2 Department of Pharmacological Sciences, Icahn School of Medicine at Mount Sinai, New York, NY 10029, USA; 3 Tisch Cancer Institute, Icahn School of Medicine at Mount Sinai, New York, NY 10029, USA; 4 Department of Pathology, Albert Einstein College of Medicine/Montefiore Medical Center, Bronx, NY 10461, USA; 5 Gruss-Lipper Biophotonics Center, Albert Einstein College of Medicine/Montefiore Medical Center, Bronx, NY 10461, USA; 6 Integrated Imaging Program, Albert Einstein College of Medicine/Montefiore Medical Center, Bronx, NY 10461, USA; 7 Medical Research Council Centre for Reproductive Health, Queen’s Medical Research Institute, University of Edinburgh, Edinburgh EH16 4TJ, UK; 8 Department of Surgery, Albert Einstein College of Medicine/Montefiore Medical Center, Bronx, NY 10461, USA

**Keywords:** breast cancer, macrophages, lung metastasis, Interleukin 4, extravasation, intravital imaging

## Abstract

**Simple Summary:**

Metastasis is the main cause of death from breast cancer. In mouse models of breast cancer lung metastasis, macrophages enhance metastasis by promoting tumor cell seeding and persistent growth. Here, we show that interleukin-4 (IL4) is required for this process as IL4 receptor (*IL4rα*)-null mice develop fewer and smaller lung metastases. This deficiency is partially rescued by adoptive transfer of wild-type monocytes. IL4 signaling in macrophages upregulates the expression of the chemokine receptor CXCR2, necessary for IL4-mediated tumor cell extravasation in vitro. In addition, expression of several other genes already causally associated with lung metastasis including *Ccl2*, *Csf1*, *Ccr1*, *Hgf* and *Flt1* are upregulated in macrophages. High-resolution intravital imaging at the time of metastatic seeding showed reduced physical interaction between tumor cells and *IL4rα*-deficient macrophages, showing the dependence on IL4. We conclude that IL4 signaling in monocytes and macrophages is important during seeding and growth of breast metastasis in the lung.

**Abstract:**

Metastasis is the systemic manifestation of cancer and the main cause of death from breast cancer. In mouse models of lung metastases, recruitment of classical monocytes from blood to the lung and their differentiation to metastasis-associated macrophages (MAMs) facilitate cancer cell extravasation, survival and growth. Ablation of MAMs or their monocytic progenitors inhibits metastasis. We hypothesized that factors controlling macrophage polarization modulate tumor cell extravasation in the lung. We evaluated whether signaling by Th1 or Th2 cytokines in macrophages affected transendothelial migration of tumor cells in vitro. Interferon gamma and LPS inhibited macrophage-dependent tumor cell extravasation while the Th2 cytokine interleukin-4 (IL4) enhanced this process. We demonstrated that IL4 receptor (*IL4rα)*-null mice developed fewer and smaller lung metastasis in E0771-LG mammary cancer models of this disease. Adoptive transfer of wild-type monocytes to *IL4rα*-deficient mice partially rescued this phenotype. IL4 signaling in macrophages controlled the expression of the chemokine receptor CXCR2, necessary for IL4-mediated tumor cell extravasation in vitro. Furthermore, IL4 signaling in macrophages regulated the transcript abundance of several other genes already causally associated with mammary cancer lung metastasis including *Ccl2*, *Csf1*, *Ccr1*, *Hgf* and *Flt1*. The central role of IL4 signaling in MAMs was confirmed by high-resolution intravital imaging of the lung in mice at the time of metastatic seeding, which showed reduced physical interaction between tumor cells and *IL4rα*-deficient macrophages. This interaction with wild-type MAMs enhanced tumor cell survival and seeding, which was lost in the *IL4rα* mice. These data indicate that IL4 signaling in monocytes and macrophages is key during seeding and growth of breast metastasis in the lung, as it regulates pro-tumoral paracrine signaling between cancer cells and macrophages.

## 1. Introduction

Breast cancer is the single most commonly diagnosed cancer among women worldwide [1,2], and it is estimated to have been responsible for the death of over 43,000 women in the US alone in 2021 [3]. The majority of cancer-related deaths, including those from breast cancer, are a result of metastatic disease and its associated complications. The fact that the survival of patients with metastatic disease has hardly improved for two decades highlights that early detection and effective treatment of established metastases is an essential clinical need.

Most experimental studies as well as sequencing studies of cancer mutations have focused on genetic and epigenetic changes that occur in cancer cells to enable metastases to prosper and occupy preferred anatomical sites. However, it has become apparent, both in primary tumors and in the seeding and expansion stages of metastatic tumors, that the environment that tumors create determines their survival and growth. This tumor microenvironment (TME), in both humans and mice, is populated by many cell types. Of these, immune cells and macrophages are highly abundant. In breast cancer, the importance of macrophages in disease progression was initially shown via genetic or pharmacological depletion in animal models, which suppressed lung metastasis [4,5]. Since then, similar studies in mouse models have also been performed in many other cancer types including glioblastoma and pancreatic cancer [6,7]. The importance of macrophages in human cancers is also supported by numerous (though not all) clinical correlative studies that have shown a correlation between high macrophage infiltration and poor prognosis [8,9,10,11]. Recently, individual macrophage transcriptional signals have also been shown to be independent prognostic indicators of poor disease-specific survival [12].

While an abundant body of information is available regarding the pro-tumoral role of macrophages at the primary site, it is still unclear whether the same mechanisms control their recruitment and activities at sites of metastases. Our previous work showed that in pulmonary foci from mouse models of metastatic breast cancer, expression of, the FMS-like tyrosine kinase 1 (FLT1, also known as Vascular Endothelial Growth Factor 1, VEGFR1), allows the identification of a particular population of macrophages (herein called metastasis-associated macrophages (MAMs)), that differ phenotypically and functionally from both tumor-associated-macrophages (TAMs) at the primary site and myeloid resident cells in the lung [13,14,15]. We showed that MAMs differentiate from circulating classical Ly6C+ CCR2+ monocytes (CMs) that are recruited to the metastases by tumor-cell-derived CCL2 [15,16]. Furthermore, once recruited, they establish direct contact with cancer cells and promote their extravasation and survival [5]. Recruitment of CMs by CCL2 is essential for metastatic progression as blocking CCL2 reduces seeding and growth of pulmonary metastasis [16]. These CMs differentiate into a MAM intermediate precursor (MAMPC) state (with properties of myeloid suppressor cells, M-MDSC) and then fully differentiate into MAMs [15]. MAMPCs respond to CCL2 by inducing the expression of CCL3 (which acts in an autocrine manner through its receptor CCR1). This promotes MAMPC retention at the metastatic site by enhancing their physical interaction with tumor cells through VCAM1. This interaction delivers a survival signal to extravasated cells and therefore enhances disease progression [17,18].

The TME in primary tumors is thought to be skewed to a Th2 type (a pro-tumoral immune response) rather than an inflammatory Th1 one (regarded as antitumoral). Supporting this hypothesis, the Th2 regulators, Interleukin-4 (IL4) and 13 (IL13), as well as IL10, have been demonstrated to play important roles during primary tumor progression in mouse models of cancer [19,20,21]. Similarly, macrophage-synthesized IL10 inhibits antitumoral cytotoxic T-cell responses, thereby promoting tumor progression. However, in this case, the mechanism appears to be via effects on dendritic cell recruitment and function, and not through TAMs [19]. In humans, high expression levels of IL4 are observed in breast tumors, which is associated with a Th2-skewed profile of tumor-infiltrating lymphocytes [22,23].

IL4R signaling can be through either a type I receptor, where the alpha chain binds to a common gamma chain (γc), or a type II receptor, where the alpha chain interacts with IL13Ra1 to form a dimer. The type II receptor allows IL13 signaling. In the TME, in addition to immune cells (myeloid and lymphoid), tumor cell lines derived from primary human breast [24,25] and lung carcinomas [26] have also been observed to have increased levels of IL4 receptors. Together, these data suggest that IL4 signaling is important for the in vivo control of tumor progression by targeting both tumor cells and immune cells in the TME.

Though much is known about primary tumors, less is known about mechanisms that regulate immunity in the metastatic site. In mouse models of bone metastasis, IL4 has also been shown to polarize bone MAMs (BoMAMs) to promote tumor growth [27]. In the lung, however, it is still unknown whether IL4 signaling participates in metastatic seeding and growth of tumor cells, or whether its role is mainly restricted to the primary tumor and bone metastases. Thus, we sought to determine whether IL4 signaling is relevant for macrophage function during lung metastatic seeding. In an experimental metastasis model that eliminates the contribution of the primary tumor, we determined that IL4 signaling in macrophages facilitates seeding of mammary tumor cells to the lung, thus suggesting a specific role for this cytokine at the metastatic site. We identified CXCR2 as an important downstream factor from IL4Rα that promotes extravasation of cancer cells in vitro. Furthermore, IL4 increases the expression of genes shown to be essential for metastatic seeding and growth, including *Ccr1*, *Flt1*, *Ccl3*, *Hgf* and *Csf1*. Importantly, high-resolution in vivo imaging of IL4rα-null mice at early stages of lung colonization demonstrates that IL4rα-null macrophages show reduced levels of physical interaction with tumor cells, which provides a mechanistic explanation for the observed reduced seeding and growth of mammary tumors in the lung.

## 2. Materials and Methods

### 2.1. Animal Models

All experiments using animals were performed in accordance with protocols approved by the Albert Einstein College of Medicine Institutional Animal Care and Use Committee (IACUC). Femurs and tibias from *Stat3^fl^*^/*fl*^ (*Tg(Csf1r-iCre)jwp^−^*^/*−*^*Stat3^flox^*^/*flox*^) and *Csf1r.iCre;Stat3^fl^*^/*fl*^ (*Tg(Csf1r-iCre)jwp^+^*^/*−*^*Stat3^flox^*^/*flox*^) were donated by Dr. Elaine Lin (Montefiore Medical Center, Bronx, NY, USA) [28]. B6-*IL4rα^tm1Sz^*/J (*IL4Rα−*/*−*) mice were donated by Dr. Johanna Joyce (Memorial Sloan Kettering Cancer Center, New York, NY, USA). Mice carrying CXCR2 homozygous deletion (C.129S2(B6)-*Cxcr2^tm1Mwm^*/J) and their WT (BALB/cJ) control were purchased from Jackson Laboratories. Femurs and tibias from animals carrying the tissue-specific CXCR2 homozygous deletion in myeloid cells (LysM-Cre recombinase x C57BL/6-*Cxcr2^tm1Rmra^*) and their Cre- controls were kindly donated by Dr. Ann Richmond (Vanderbilt University). MacBlue (B6,FVB-Tg(Csf1r-GAL4/VP16,UAS-ECFP)1Hume/J) mice [29] were purchased from Jackson Laboratories. WT FVB/NJ mice were also purchased from Jackson laboratories. All animals were housed in SPF conditions.

### 2.2. Cell Lines and Tissue Culture

Mouse E0771 medullary mammary adenocarcinoma cells were obtained from Dr. E. Mihich (Rosewell Park Cancer Institute, New York, NY, USA). E0771 cells were originally isolated from a spontaneous mammary tumor in C57BL/6 mice [30]. Highly metastatic lung-tropic E0771 tumor cells (E0771-LG) were previously derived from the parental cell line by isolation from lung foci after repeated cycles of experimental metastasis assays [18]. Murine endothelial 3B-11 cells were obtained from the ATCC^R^ (CRL-2160^TM^). Met-1 cells were derived from a spontaneous mammary carcinoma from the MMTV-PyMT mouse model in FVB mice [31]. All cell lines were maintained in Dulbecco’s modified Eagle medium (DMEM) (Invitrogen, Carlsbad, CA, USA) supplemented with GlutaMAX^TM^-I, 10% *v*/*v* heat-inactivated fetal bovine serum (FBS), 1 mM sodium pyruvate, 100 U/mL penicillin and 100 µg/mL streptomycin at 37 °C and 5% *v*/*v* CO_2_ (complete DMEM). The fluorescent E0771-LG cell line expressing Clover (Addgene) [32] used in intravital experiments was established by standard transfection, as previously published. To obtain these, E0771-LG cells were cultured overnight in 10% *v*/*v* FBS-DMEM without antibiotics and transfected with lipofectamine^®^ 2000 together with the CLOVER plasmid. Cells were cultured in selection media (700 µg/mL G418 in complete DMEM) for a week and enriched for the 10% brightest population using fluorescence-activated cell sorting (FACS). Sorted cells were maintained under selection for a second week and enriched once again for the 10% brightest population by FACS. The cell line established was evaluated for compatibility with intravital imaging (brightness, photobleaching and photodamage). E0771-LG cells expressing Clover were subjected to a third round of selection in which single-cell clones were isolated in 96-well plates by FACS. All intravital imaging sessions were performed using two clones, #14 and #15.

### 2.3. Primary Culture of Bone-Marrow-Derived Macrophages

Femurs and tibias from 8–12-week-old mice were collected and flushed with a 23 g needle in cold Alpha Minimum Essential (αMEM) Medium. The bone marrow cell suspension was centrifuged at 180 g for 5 min and plated overnight in tissue culture plates in 10% *v*/*v* heat-inactivated FBS, 100 U/mL penicillin, 100 µg/mL streptomycin and 10^4^ U/mL human recombinant (rh) CSF1 (complete αMEM) at 37 °C and 5% *v*/*v* CO_2_. Non-adherent cells were then transferred to Petri dishes and cultured in complete αMEM for 6 days. Cells were supplemented with fresh complete αMEM every 3 days and used for experiments no later than 2 weeks after bone marrow isolation.

### 2.4. Endothelial Cell Permeability Assay and Transendothelial Resistance

Matrigel(R)-coated transwell inserts (Corning, New York, NY, USA) with 8 µm pores were thawed at room temperature for 10 min and pre-incubated with complete DMEM in both chambers for two hours at 37 °C. Then, 10^4^ 3B-11 cells were seeded on top of the filter in complete DMEM at different time points. For transendothelial resistance (TER) assays, the medium was replaced with DMEM containing 0.5% *v*/*v* FBS, 1 mM sodium pyruvate, 100 U/mL penicillin and 100 μg/mL streptomycin (0.5% *v*/*v* FBS-DMEM) two hours before measurement. Transendothelial resistance was measured using an EndOhm-6 chamber and EVOM^2^ resistance meter (World Precision Instruments, Sarasota, FL, USA) and expressed as a function of the effective surface area of the filter membrane (Ω × cm^2^). For permeability assays, media in the receiving (top) chambers was replaced with DMEM containing 0.5% *v*/*v* FBS, 1 mM sodium pyruvate, 100 U/mL penicillin, 100 μg/mL streptomycin and 100 µg/mL of 70 kDa Rhodamine B Isothiocyanate–Dextran (TRITC) and incubated for 2 h. Aliquots of 100 µL from the receiving and bottom chambers were taken and diluted at 1:5 in DMEM before reading using a SpectraMax M5 plate reader (emission/excitation = 557/586 nm; Molecular Devices). Concentrations were calculated using a standard curve. Control condition consisted of transwell inserts with no 3B-11 cells, incubated for 2 h in 0.5% *v*/*v* FBS-DMEM.

### 2.5. Experimental Transendothelial Migration Assay

Matrigel(R)-coated transwell inserts (Corning) with 8 µm pores were thawed at room temperature for 10 min and pre-incubated with complete DMEM in both chambers for two hours at 37 °C. Then, 10^4^ 3B-11 murine endothelial cells were seeded in 200 µL of complete DMEM in the receiving chamber (top), with 750 µL of complete DMEM in the bottom chamber. Cells were incubated for 48 h at 37 °C and 5% *v*/*v* CO_2_ until confluent. E0771-LG cells and Met-1 cells were labeled with CellTracker^TM^ Green CMFDA Dye (Thermo Fisher Scientific, Princeton, NJ, USA) following the manufacturer’s instructions, trypsinized and adjusted to 1 × 10^5^ cells/mL (Met-1 cells) or 2 × 10^5^ cells/mL (E0771-LG cells) in 0.5% *v*/*v* FBS-DMEM containing 10^4^ U/mL hrCSF1. Bone-marrow-derived macrophages (BMDMs) were lifted into a cell suspension with a rubber policeman and adjusted to 5 × 10^5^ cells/mL in complete DMEM supplemented with 10^4^ U/mL of rhCSF1. Macrophages were seeded on the bottom side of the transwell insert by carefully depositing 20 µL of the cell suspension and allowing attachment for 30 min at room temperature. Transwell inserts were moved to wells containing 750 µL of complete DMEM with 10^4^ U/mL of rhCSF1 and received 200 µL of tumor cell suspension in the top chamber. After 36 h, cells at the receiving chamber were removed using a cotton swab. Cells at the bottom chamber were fixed in 4% *w*/*v* paraformaldehyde (PFA) in PBS for 30 min at 4 °C. Six randomly selected fields were acquired per transwell insert in an inverted Olympus IX81 microscope equipped with a 20X (NA = 0.40 Air) objective and a Sensicam QE cooled CCD camera (Analytical Imaging Facility, Albert Einstein College of Medicine). Images were quantified using Fiji/ImageJ software [33]. Each experiment was performed in a minimum of three independent repeats, and in triplicate. BMDMs, unless otherwise specified, were stimulated with cytokines for 24 h at 10 ng/mL. Recombinant murine IL4, IL13, IL10, CXCL1, CXCL2 and CXCL5 were purchased from Peprotech and reconstituted in sterile 0.05% *w*/*v* BSA-PBS and stored at −80 °C. Blocking antibody against IL10R was used at 20 ng/mL (Rat anti-mouse CD210, BD#550012). CXCR2 inhibitor SB332235 (GlaxoSmith, Durham, NC, USA) was diluted in DMSO and used at 100 µM in culture medium.

### 2.6. Experimental Metastasis Assay

Female mice 8–12 weeks in age were intravenously injected with 6 × 10^5^ syngeneic tumor cells through the tail vein in a total volume of 200 µL of PBS as described [18]. Injected mice were maintained under quarantine until tissue collection. Mice were euthanized by isoflurane overdose, and lungs were perfused with 5–10 mL of PBS injected to the right ventricle of the heart. Lungs were filled with 1 mL of 4% *w*/*v* PFA in PBS and fixed overnight in the same fixative. Metastatic burden was assessed by serial sectioning of PFA-fixed paraffin-embedded lung tissue sections stained with hematoxylin and eosin (H&E) using the stereological method [34]. The metastatic (Met) index was calculated as the metatasis volume nomalized to lung volume = total area of foci / total area of the lung and represents the total tumor burden that includes foci number and foci size [5,34].

### 2.7. Adoptive Transfer Experiments

Donor female mice 8–12 weeks in age were euthanized by isoflurane overdose and femurs, tibias, ileac bone and spine were collected to isolate bone marrow cells. Bones were crushed using mortar and pestle in sterile FACS buffer (1% *w*/*v* BSA in PBS) and filtered through a 40 µm cell strainer to obtain single-cell suspensions. Cell suspensions were centrifuged at 280 g and 4 °C for 5 min, and erythrocytes were eliminated by incubation in RBC buffer for 10 min on ice. Cells were counted and adjusted to 2.8 × 10^8^ cells/mL in MACS buffer (1X PBS, 0.5% *v*/*v* FBS, 2 mM EDTA). Monocytes were enriched by negative selection using a Monocyte Isolation Kit (Miltenyi Biotech, Gaithersburg, MD, USA, Kit #130-100-629) following the manufacturer’s instructions. After magnetic separation, inflammatory monocytes (CD45^+^CD11b^+^Ly6G^−^Ly6C^+^) were collected using a FACS Aria unit (Becton Dickinson, Franklin Lakes, NJ, USA) in sterile conditions. Female mice 8–12 weeks in age were intravenously injected with 6 × 10^5^ EO771-LG tumor cells mixed in a 1:1 ratio with monocytes through the tail vein in a total volume of 200 µL of PBS as described before [18]. Injected mice from three different cohorts were maintained under quarantine for 11 days before tissue collection.

### 2.8. Real-Time PCR

Total RNA was isolated from cells by homogenization in TRIzol (Thermo Fisher) or RNeasy miniprep kit (Qiagen, Germantown, MD, USA) and converted into cDNA by using SuperScript^®^ Vilo (Thermo Fisher). Gene expression was determined by real-time PCR using SyBRGreen (Invitrogen) and is shown as relative gene expression after normalization to *GAPDH*. The following primers were used: *Gapdh* forward: 5′-CTTTGTCAAGCTCATTTCCTGG-3′; *Gapdh* reverse: 5′-TCTTGCTCAGTGTCCTTGC-3′; *Cxcl2* forward: 5′-AATGCCTGAAGACCCTGC-3′; *Cxcl2* reverse: 5′-TTTTGACCGCCCTTGAGAG-3′; *Ccl3* forward: 5′-ATGAAGGTCTCCACCACTGC-3′; *Ccl3* reverse: 5′-CCCAGGTCTCTTTGGAGTCA-3′; *Ccl5* forward: 5′-GTTCCATCTCGCCATTCATG-3′; *Ccl5* reverse: 5′-TTAAGCAAACACAACGCAGC-3′; *Cxcl1* forward: 5′-AGAACATCCAGAGCTTGAAGG-3′; *Cxcl1* reverse: 5′-CAATTTTCTGAACCAAGGGAGC-3′; *Cxcr2* forward: 5′-CGTTTGAGGGTCGTACTGCG-3′; *Cxcr2* reverse: 5′-TGGGCCTTAAAGAGGGTGCG-3′; *Flt1* forward: 5′-GAGCCAGGAACATATACACAGG-3′; *Flt1* reverse: 5′-GCTTGACAGTCTAAGGTCGTAG-3′; *Vegf1* forward: 5′-AAAGCCAGCACATAGGAGAG-3′; *Vegf1* reverse: 5′-ATTTAAACCGGGATTTCTTGCG-3′; *Il13* forward: 5′-CCAGCCCACAGTTCTACAG-3′; *Il13* reverse: 5′-GAGACACAGATCTTGGCACC-3′.

### 2.9. Ingenuity Pathway Analysis

Datasets comparing BMDMs treated with recombinant murine IL4 for 24 h at 20 ng/mL and untreated control conditions were accessed from the NCBI database. Gene list with accession number GSE35435 [35] was uploaded to Ingenuity Pathway Analysis tool (Qiagen) and curated for differentially expressed genes (*p*-value < 0.05) and fold change > 1.5.

### 2.10. Flow Cytometry

Blood samples were collected through cardiac puncture or retro-orbital bleeding in heparin. Single-cell suspensions were incubated for 10 min in red blood cell (RBC) lysis buffer (Biolegend, San Diego, CA, USA) on ice to eliminate erythrocytes. Cell suspensions were incubated for 30 min on ice with anti-mouse Fc Block CD16/32 antibody (eBioscience, San Diego, CA, USA) in FACS buffer to avoid non-specific antibody binding. Cells were then washed in FACS buffer and stained with either Ig controls or fluorophore-conjugated antibodies in FACS buffer. Data acquisition was performed on a LSR II Yellow (BD Biosciences, San Jose, CA, USA) and analyzed on FlowJo version 10 (FlowJo, LLC, Ashland, OR, USA). Classical (CM: CD45^+^ CD11b^+^ F4/80^+^ Ly6G^−^ Ly6C^hi^) or non-classical patrolling monocytes (PM: CD45^+^ CD11b^+^ F4/80^+^ Ly6G^−^ Ly6C^−^) were identified by FACS using the above antibodies as described [16]. Antibodies used were: anti-CD45-PerCpCy5.5 (eBioscience #45-0451-82); CD11b-eVolve 605 (eBioscience #83-0112-42); F4/80-FITC (Biolegend #123108); Ly6C-PE-Cy7 (Biolegend #128018); Ly6G-APC-Cy7 (Biolegend #127624); CD115-APC (eBioscience #17-1152-82); CD11b-PECy7 (Biolegend #101216); Ly6C-APC-Cy7 (BD-560596); Ly6G-PE (BD#551461).

### 2.11. Intravital Imaging

Female WT or *IL4rα*^−/−^ MacBlue mice 6–12 weeks in age were prepared for surgery as previously described [36,37]. Mice were connected to a mechanical ventilator (Harvard Apparatus, Holliston, MA, USA) providing 0.2 cc of air at ~140 cycles per second and 3% *v*/*v* of isoflurane. Then, skin and muscle layers of the upper left thorax were removed. The largest lung lobule was exposed by resection of ribs and intercostal muscles. A vacuum-stabilized lung window [38] was attached to the exposed lung area. Mice were maintained under light anesthesia at physiological temperature and hydrated by periodic injections of saline. Vital signs were monitored using a pulse oximeter (MouseOx, Starr LifeSciences, Oakmont, PA, USA). Vasculature was labeled with an intravenous injection of 155 kD TRITC Dextran (Sigma-Aldrich, Burlington, MA, USA). Imaging sessions were performed with intravenous delivery of up to 2 × 10^6^ Clover-expressing E0771-LG cells in *IL4rα*^+/+^ MacBlue or *IL4rα*^−/−^MacBlue mice. Imaging was performed on a custom-made 2-laser-2-photon microscope (Gruss Lipper Biophotonics Center, Albert Einstein College of Medicine) [38]. At the end of imaging sessions, animals were euthanized by isoflurane overdose and cervical dislocation. The high-resolution images were analyzed using ImageJ. The location of tumor cells with an imprecise location regarding the vasculature was determined to be in the intravascular or extravascular space by fluorescence signal intensity in the dextran (red) channel to determine blood serum exclusion over an 8 um region of interest (ROI) on the tumor cell, on a perfused vessel and a background area, as previously described. The tumor cell interaction with monocytes or macrophages was considered positive where the green/Clover signal and myeloid CFP signal were immediately adjacent or overlapping in adjacent planes. The time of interaction was calculated by the factor of interval between frames and the number of consecutive frames where green/Clover and CFP signals were adjacent/overlapping. For cell tracking, the tumor cell green signal was cleaned up from the overlapping CFP signal by subtraction and thresholded to define cell outlines. Tracking was performed over outlines of tumor cells and myeloid cells using a custom-made plugin for ImageJ called ROI_Tracker [38]. The number of contacts between tumor cells and monocytes was determined by the number of times the green and cyan signal were immediately adjacent or overlapping within the same space (intravascular) over 30 consecutive frames and then expressed as contacts per hour. The number of monocytes contacting tumor cells per hour was determined by counting the number of new cells in contact with one tumor cell within 30 frames.

### 2.12. Statistical Analysis

Comparisons between two groups were evaluated by Student’s unpaired *t*-test. Comparisons between multiple groups were evaluated by one-way analysis of variance (ANOVA) with Dunnett correction for multiple comparisons. Comparisons between groups for in vivo experiments were evaluated by the Mann–Whitney or Kruskal–Wallis test. Adjusted *p*-values are shown, unless otherwise indicated. For in vitro eTEM assays, normalization against the value of transendothelial migrated tumor cells in the presence of WT or untreated BMDMs was used to express differences as percentages. All statistical analyses were performed using the Prism-GraphPad software.

## 3. Results

### 3.1. Interleukin 4 Signaling in Macrophages Enhances Cancer Cell Transendothelial Migration

**Figure 1:**
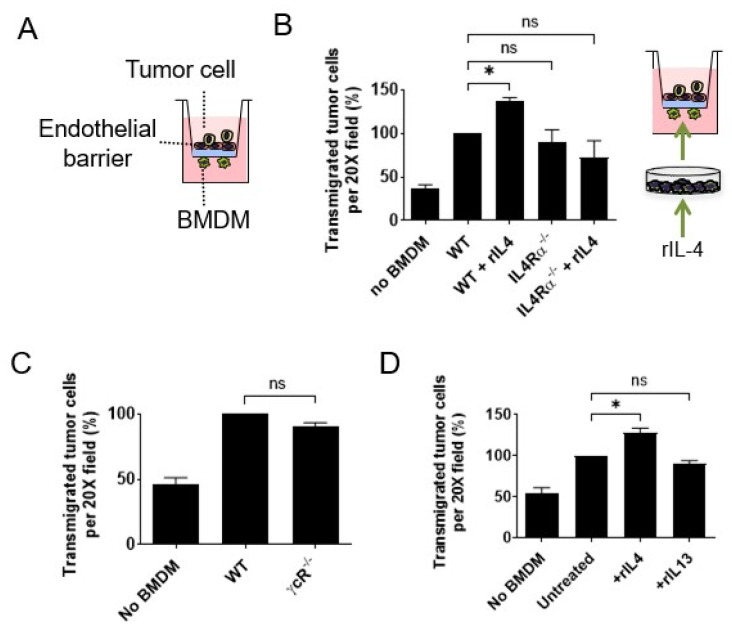
IL4 promotes macrophage-mediated tumor cell transendothelial migration. (**A**) Schematics of the transendothelial migration assay modeling extravasation (eTEM). An endothelial monolayer (red) sits over a Matrigel-coated transwell. Endothelial barrier is exposed to tumor cells on the apical domain. Macrophages are added to the bottom side of the transwell insert (closer to the basal domain of the endothelial barrier). Tumor cells that migrate through the endothelial layer and matrigel are quantified once present in the bottom side of the transwell insert. (**B**) Transendothelial migration of E0771-LG cells in response to WT or *IL4rα-null* BMDMs pre-incubated with vehicle or rIL4. ANOVA and Dunnett correction, *n* = 3, * *p*-value < 0.05. (**C**) Transendothelial migration of Met-1 cells in presence of BMDM deficient for the IL4R co-receptor γ_C_R. Mann–Whitney test, *n* = 3, *p*-value > 0.1. (**D**) Transendothelial migration of Met-1 cells in response to WT BMDMs pre-incubated with recombinant IL4 or IL13 for 24 h. Mann–Whitney test, *n* = 3, * *p*-value < 0.01. Data are presented as the mean normalized to WT or untreated control +/− SD. Each experiment was performed in duplicate or triplicate, and each n corresponds to an independent experiment performed with BMDM from independent mice.

In vitro assays that mimic extravasating tumor cell transendothelial migration (eTEM) at distant sites have been useful for identifying the signaling pathways by which macrophages promote extravasation in vivo activities with high fidelity (Figure 1A) [5,16,39]. Therefore, we utilized this eTEM assay to evaluate whether Th2 or Th1 polarization plays a role in regulating macrophage promotion of tumor cell transendothelial cell migration. In order to study mammary cancer metastasis, the transendothelial migration of the tumor cell lines Met-1 (derived from a PyMT mammary tumor) and E0771-LG (derived from a medullary mammary cancer selected for high metastatic potential) in response to bone-marrow-derived macrophages (BMDMs) was analyzed as previously described [39]. To confirm the presence of a tight endothelial barrier in the assay, we measured the permeability of the endothelial layer using both 10 and 70 kDa dextrans as well as the electrical resistance across the barrier (Supplemental Figure S1A,B), as previously described [40]. After this eTEM assay optimization, IL4Rα signaling was analyzed. First, the effect of an exogenous source of IL4 on tumor cell transendothelial migration events in vitro in the presence of macrophages was measured. Wild-type (WT) BMDMs were pre-incubated with recombinant IL4 and placed into the assay. This IL4 treatment induced a significant increase in the number of E0771-LG cells (*n* = 3, <0.005) (Figure 1B) and Met-1 (Supplemental Figure S1C, *n* = 3, *p*-value < 0.0001) cells that migrated across the endothelial barrier compared to untreated controls. This response was specific to IL4 signaling, as IL4 stimulation of BMDMs isolated from *IL4Rα^−^*^/*−*^ mice did not enhance tumor cell transendothelial migration (Figure 1B; *n* = 3, *p*-value > 0.05). However, in contrast to this enhancement by IL4, there was no significant difference in the number of either Met-1 or E0771-LG tumor cells that transmigrated the endothelial barrier in response to *IL4Rα^−^*^/*−*^ BMDMs compared to WT BMDMs (Met-1 cells, *n* = 5, *p*-value_(*IL4Rα+*/*−*)_ = 0.59 and *p*-value _(*IL4Rα−*/*−*)_ = 0.15; E0771-LG cells, *n* = 4, *p*-value_(*IL4Rα+*/*−*)_ = 0.88 and *p*-value_(*IL4Rα−*/*−*)_ = 0.98) (Supplemental Figure S1D). Similarly, BMDMs isolated from mice lacking the γ_C_ receptor, a subunit that is required for the formation of the type I IL4R complex, did not alter the transendothelial migration of Met-1 cells (Met-1 cells, *n* = 3, *p*-value = 0.14) (Figure 1C). Thus, IL4 signaling was not required for macrophage-enhanced tumor cell transendothelial migration in this assay, but IL4 potentiated the macrophage activity. To evaluate whether IL13 (a cytokine sharing the type II IL4R) mediates a similar response, BMDMs were stimulated with recombinant IL13, and the tumor cell transendothelial migration was measured. In contrast to IL4, there was no change in the number of transmigrated tumor cells after pre-stimulation of BMDMs with IL13 (Met-1 cells, *n* = 3, *p*-value = 0.33) (Figure 1D).

Macrophages in the primary tumor TME can synthesize IL10, another Th2 cytokine [19]. Thus, to study IL10 signaling in macrophage promotion of tumor cell transendothelial migration, eTEM assays were used with BMDMs with and without a neutralizing antibody for the IL10 receptor. The IL10-receptor-inhibitory antibody was applied either in the bottom or both chambers in the absence of exogenous IL10. This inhibitory antibody had no effect on the promotion by BMDMs’ of Met-1 transendothelial migration (Supplemental Figure S1D). To further investigate IL10 signaling, BMDMs were pre-incubated with or without IL10 and compared in the eTEM assay. However, this IL10 treatment made no significant difference in their activity compared to untreated BMDMs in the eTEM assay (Supplemental Figure S1E). IL10 signals via the signal transducer and activator of transcription (STAT3), the central transcriptional effector in macrophages. *Stat3*-null mutant mice are not viable. Thus, to study the dependence on STAT3 in the eTEM assay, BMDMs were derived from a homozygous floxed allele for *Stat3* crossed onto a myeloid-restricted *Csf1* receptor (*Csf1r)* improved cre recombinase strain [13,28]. BMDMs when *Stat3* was deleted by the presence of the *Csf1r-icre* did not have significantly different promotion activity compared with those derived from either WT or *Stat3^fl^*^/*fl*^ mice (Supplemental Figure S1F). We can thus conclude that IL10-STAT3 signaling does not affect macrophage stimulation of tumor cell transendothelial migration in vitro.

To assess the role of Th1 cytokines and stimulators of macrophage activation in the eTEM assay, BMDMs were treated with IFNgamma, the TLR4 agonist lipopolysaccharide (LPS) or both together. In all cases, these treatments completely abolished the macrophage stimulation of tumor cell transendothelial cell migration to the basal level achieved by tumor cells alone (Supplemental Figure S1G).

Together, these results show that WT BMDMs promote tumor cell transendothelial migration in vitro while IL4Rα signaling from IL4 (but not other Th2 or Th1 cytokines) in macrophages enhances their activity in this process. These in vitro data led us to hypothesize that the lung metastatic TME is aided by Th2 polarization at the seeding step.

### 3.2. IL4Rα Is Important for Efficient Lung Metastasis Development

**Figure 2:**
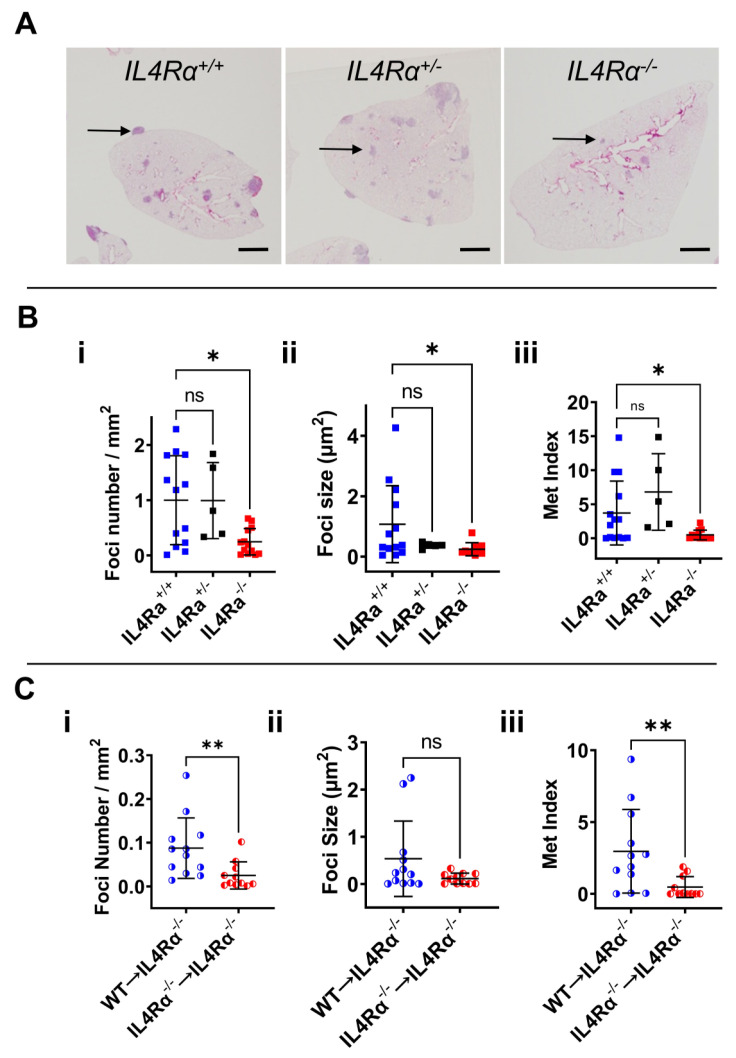
IL4 signaling is important for pulmonary metastatic colonization. (**A**) Hematoxylin-and-eosin-stained sections of the lungs from WT, *IL4Rα*^+/−^ and *IL4Rα*^−/−^ mice intravenously injected with syngeneic E0771-LG cells 11 days post-injection. Scale bar = 1 mm. (**B**): (**i**) Stereological quantification of foci number in WT, *IL4Rα*^+/−^ and *IL4Rα*^−/−^ mice 11 days after intravenous injection of E0771-LG cells by counting the number of all foci every 100 µm interval in a total of six sections. Graphs correspond to 5 to 13 mice per group with bars representing average +/− SD. Kruskal–Wallis test, * *p*-value < 0.05. (**ii**) Stereological quantification of foci size in WT, *IL4Rα*^+/−^ and *IL4Rα*^−/−^ mice 11 days after intravenous injection of E0771-LG cells, calculated after measuring the area of all foci every 100 µm interval in a total of six sections. Graphs correspond to 5 to 13 mice per group with bars representing average +/− SD. Kruskal–Wallis test, * *p*-value < 0.05. (**iii**) Stereological quantification of the metastatic index, the ratio of total foci area by lung area, in WT, *IL4Rα*^+/−^ and *IL4Rα*^−/−^ mice 11 days after intravenous injection of E0771-LG cells. Graphs correspond to 5 to 13 mice per group with bars representing average +/− SD. Kruskal–Wallis test, * *p*-value < 0.05. (**C**): (**i**) Stereological quantification of foci number in *IL4Rα^−^*^/*−*^ mice adoptively transferred with WT (WT to *IL4Rα*^−/−^) or *IL4Rα*^−/−^ (*IL4Rα*^−/−^ to *IL4Rα*^−/−^) monocytes immediately after E0771-LG cell injection. Graphs correspond to 11 to 12 mice per group with bars representing average +/− SD. Mann–Whitney test, *n* > 11/group, ** *p*-value < 0.005. (**ii**) Stereological quantification of foci size in *IL4Rα^−^*^/*−*^ mice adoptively transferred with WT (WT to *IL4Rα*^−/−^) or *IL4Rα*^−/−^ (*IL4Rα*^−/−^ to *IL4Rα*^−/−^) monocytes immediately after E0771-LG cell injection. Graphs correspond to 11 to 12 mice per group with bars representing average +/− SD. Mann–Whitney test, *n* > 11/group, *p*-value > 0.05. (**iii**) Stereological quantification of metastatic index in *IL4Rα^−^*^/*−*^ mice adoptively transferred with WT (WT to *IL4Rα*^−/−^) or *IL4Rα*^−/−^ (*IL4Rα*^−/−^ to *IL4Rα*^−/−^) monocytes immediately after E0771-LG cell injection. Graphs correspond to 11 to 12 mice per group with bars representing average +/− SD. Mann–Whitney test, *n* > 11/group, ** *p*-value < 0.005. Blue square or circles are WT mice, black squares are *IL4Rα*^+/−^ mice, red squares or circles are *IL4Rα*^−/−^ mice.

In previous studies, static imaging methods of the whole metastatic lung enabled the visualization of cells in the process of extravasation. These data show that macrophages stimulate tumor cell extravasation [5]. Data are consistent with the in vitro transendothelial migration assay above. Once tumor cells have extravasated, for them to grow, they first need a survival signal as this process, at least in experimental metastasis models, is very inefficient [38]. Studies have shown that this survival signal is delivered by MAMs [16,17,18]. The efficiency of initial growth can be measured using quantitative stereological methods that discriminate focal numbers and their subsequent growth. We used this method to test this hypothesis that IL4 drives metastasis in vivo, and an experimental metastasis assay using tail vein injections of syngeneic E0771-LG cells was employed to compare disease load in *IL4Rα*-null mutants compared to WT mice, both on a BL6 background. Lungs were collected 11 days after injection and processed for metastatic burden quantification (Figure 2A). Rigorous stereological quantification of tumor burden in the lungs [5] showed that *IL4Rα*-null mice developed fewer foci (Figure 2(Bi)) (*n* = 13, *p*-value < 0.005) with decreased size (Figure 2(Bii)) compared to WT mice (*n* = 13, *p*-value < 0.05), indicative of reduced seeding and persistent growth of cancer cells, respectively. The metastatic index that indicates total metastatic load, was also decreased in *IL4Rα^−^*^/*−*^ mice (*n* = 13, *p*-value < 0.05) (Figure 2(Biii)). Although there was a trend towards a reduced foci number, foci size and metastatic index in heterozygous mice (*IL4Rα^+^*^/*−*^*)* compared to WT mice, this reduction did not reach statistical significance when comparing WT and *IL4Rα^+^*^/*−*^ mice or *IL4Rα^+^*^/*−*^ and *IL4Rα^−^*^/*−*^ mice (Figure 2(Bi,Bii,Biii)). These results indicate that IL4 signaling through *IL4Rα* is important for tumor cell seeding and growth in vivo.

Initial seeding of metastatic cells is enhanced by classical monocytes [16]; thus, we assessed if their number was reduced in the *IL4Rα^−^*^/*−*^ mice. No significant difference in the number of circulating classical (CM: CD45^+^ CD11b^+^ F4/80^+^ Ly6G^−^ Ly6C^hi^) or non-classical patrolling monocytes (PM: CD45^+^ CD11b^+^ F4/80^+^ Ly6G^−^ Ly6C^−^) from the total CD45^+^ CD11b^+^ F4/80^+^ cells was observed between WT and *IL4Rα^−^*^/*−*^ mice (*n* > 9, *p*-value > 0.15) (Supplementary Figure S2A,B). Thus, the absence of *IL4Rα* did not affect the number of circulating monocytes available to be recruited by tumor cells to the lungs and, consequently, the reduced tumor burden in the lung of *IL4Rα^−^*^/*−*^ mice was not due to a reduction in the availability of circulating monocytes but their function.

The null mutation in *IL4Rα* mice affects signaling in all cell types where it is expressed. Thus, to determine if IL4 signaling in monocytes plays a part in the stimulation of lung metastasis by IL4, classical monocytes (CMs) were isolated from WT or *IL4Rα^−^*^/*−*^ mice and adoptively transferred into *IL4Rα*-null mice (WT to *IL4Rα^−^*^/*−*^*; IL4Rα^−^*^/*−*^ to *IL4Rα^−^*^/*−*^), and the effect on metastasis was evaluated. We observed that WT CMs increased the foci number (Figure 2(Ci)) and the total metastatic burden (metastatic index) (Figure 2(Ciii)) in *IL4Rα^−^*^/*−*^-null mice while *IL4Rα^−^*^/*−*^ CMs did not (*n* > 11, *p*-value 0.012). In these experiments, focal size (Figure 2(Cii)) was not significantly restored, probably due to the short life span of the adoptively transferred CMs that were only transferred once with the cancer cells at the beginning of the assay. Nevertheless, these gain-of-function over loss-of-function results conclusively show that a deficiency in IL4 signaling in monocytes/macrophages is at least a significant part of the cause for the reduced metastatic burden in *IL4Rα* null mice.

### 3.3. IL4 Signaling Stabilize Tumor Cell–Macrophage Contact In Vivo

**Figure 3:**
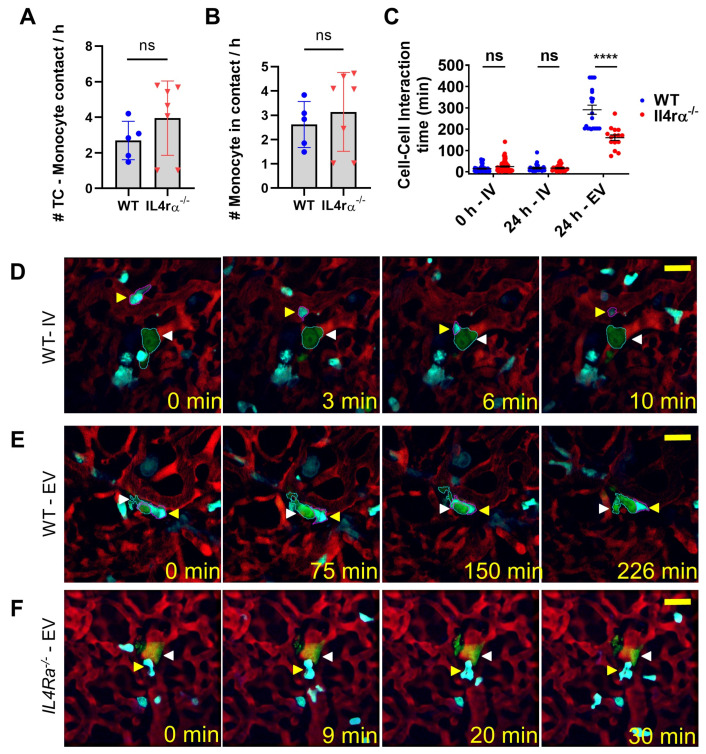
Tumor cell–macrophage interaction in the pulmonary metastatic site in vivo. (**A**) Quantification of the number of physical contacts between a tumor cell and monocytes per hour immediately after tumor cell arrival at the lungs. Mann–Whitney test, ns non-significant. (**B**) Quantification of the number of new monocytes contacting a tumor cell per hour. Mann–Whitney test, ns non-significant. (**C**) Quantification of the time of physical interaction between tumor cells (green) and monocytes (intravascular CFP+ cells) or macrophages (extravascular CFP cells). Two-way ANOVA with Bonferroni post-test, ns non-significant, **** *p*-value < 0.0001. Blue and red symbols WT and *IL4Rα*^−/−^ mice, respectively. (**D**) Two-photon microscopy of the lungs of WT MacBlue mice, shortly after (0 h post-injection) intravenous injection of Clover^+^ E0771-LG cells. Tumor cell (green, white arrowhead) interacts with CFP^+^ monocyte (cyan, yellow arrowhead) in the intravascular space (vasculature lumen labeled in red by injection of TRITC-Dextran). (**E**) Two-photon microscopy of the lungs of WT MacBlue mice 24 h after intravenous injection of Clover^+^ tumor cells. An extravascular tumor cell (green, white arrowhead) is seen physically interacting with CFP^+^ macrophage (cyan, yellow arrowhead). (**F**) Two-photon microscopy of the lungs of *IL4Rα^−^*^/*−*^ MacBlue mice 24 h after intravenous injection of Clover^+^ tumor cells. An extravascular tumor cell (green, white arrowhead) is physically interacting with CFP+ cell (cyan, yellow arrowhead). For D-F, scale = 40 um. For Figures A and B, 11 EO771-LG Clover cells from 4 Il4Ra-null mice were quantified, and 8 EO771-LG clover cells from 3 WT animals were quantified. For Figure C, tumor cells from 3 WT and 5 *Il4Ra*-null mice were quantified.

Previous data indicate that CCL3/CCR1 signaling regulates tumor cell macrophage interactions that, once established, deliver a survival signal to the tumor cells through engagement of αV-integrin expressed on MAMs and VCAM1 on the tumor cell. This enhances metastatic cell seeding capacity as inhibition of this signaling pathway or of monocyte recruitment inhibited metastases [16,17,18]. Here, we used a novel multiphoton intravital imaging of the lung with a vacuum-stabilized imaging window [36,37] coupled with quantitative measurements to elucidate the dynamics of monocyte recruitment and differentiation (into MAMs), as well as their interaction with tumor cells during tumor cell lodging and seeding. In previous reports using MacBlue mice in which monocytes and macrophages were labeled with CFP [29,36], we could phenotypically identify monocytes as small CFP+ cells in the circulation and differentiate them from tissue-resident macrophages, which showed static behavior and an extravascular location [37]. We previously showed that even single-cell-stage tumor foci were characteristically associated with macrophages and that there was a tight physical interaction with macrophages without signs of tumor cell death [36]. In the current experiments, metrics were developed based upon cell tracking and were used to quantify the number of tumor cell–monocyte and tumor cell–macrophage interactions in WT and *IL4rα* mutant mice. Immediately after arrival of tumor cells at the lungs (0–1 h post-injection), we observed that individual tumor cells arrested in the lungs had contact with monocytes at an average per hour of 2.6 ± 1 times in WT animals and 3.9 ± 2 times in *IL4rα*-null animals, with no significant difference between groups (*p* < 0.32) (Figure 3A). We also determined the number of contacts between tumor cells and new monocytes (monocytes that have never interacted with arrested tumor cells before). There was no statistically significant difference between WT vs. *IL4rα*-null animals (2.62 ± 1 in WT and 3.1 ± 2 in *IL4rα, p* = 0.50) (Figure 3B). Then, we quantified the time of tumor cell–monocyte and tumor cell–macrophage interactions in WT and *IL4rα* mutant mice. Upon arrival in the lung of WT mice, intravascular tumor cells had limited interactions with circulating monocytes, lasting an average of 14 ± 2 min (*n* = 50). In *IL4rα*-null mutant mice, the initial interactions between tumor cells and monocytes were similar to those observed in WT mice, with an average interaction time of 20.3 ± 3 min (*n* = 46, *p* > 0.05) (Figure 3C,D; Supplemental Video S1). Later, 24 h post-injection, the fraction of tumor cells that remained in the intravascular space showed a similar number of interactions with monocytes (Figure 3C for WT and *IL4rα*-null at 24 h,). However, in WT mice, as soon as tumor cells had extravasated (Figure 3C), they displayed stable physical contact with macrophages that lasted an average of 195 ± 30 min (*n* = 25), significantly longer than tumor cell–monocyte interactions (*p* < 0.0001) (Figure 3C,E; Supplemental Video S2). This is also likely an underestimate of the time because our animal protocol did not allow imaging times over 8 h. Importantly, this time of interaction of tumor cells with *IL4rα*^−/−^ macrophages, whilst longer than initial intravascular interactions, was significantly shorter than in WT mice, with an average of 84.13 ± 12 min (*n* = 43, *p* < 0.0001) (Figure 3C,F; Supplemental Video S3). Thus, IL4 signaling maintained the stability of MAM–tumor cell interactions but not their initial interactions with monocytes. These data together with the monocyte rescue results above indicate a significant role for IL4 signaling via monocyte/macrophages in metastatic cell seeding and survival.

### 3.4. CXCR2 Is Downstream of IL4 in Macrophages and Mediates the IL4-Dependent Increase in Tumor Cell Transendothelial Migration

**Figure 4:**
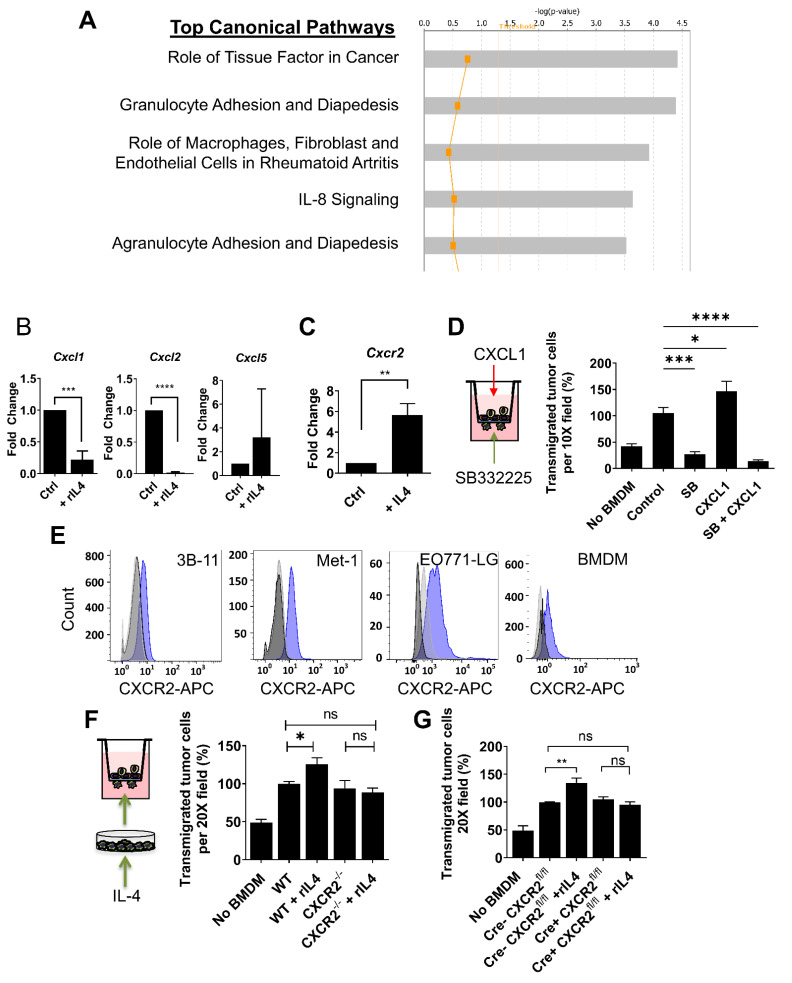
CXCR2 is controlled by IL4 signaling in macrophages, and it mediates the IL4-dependent transendothelial migration of tumor cells. (**A**) Top 5 canonical pathways derived from ingenuity pathway analysis (IPA) for differentially expressed genes in BMDM stimulated with IL4. These pathways emerged following IPA “Core Analysis.” Graph shows category scores (y axis) based on the *p*-values derived from the Fisher’s exact test. The x axis displays the –log(*p*-value). The yellow threshold line represents a significance cutoff at *p* = 0.05. (**B**) Normalized level of mRNA expression measured by qPCR of IL-8 functional homologs CXCL1, CXCL2 and CXCL5 in macrophages after a 24 h stimulation with recombinant IL4 (*n* = 3, *** *p*-value <0.001; **** *p*-value < 0.0001). (**C**) Normalized level of expression of the mRNA *Cxcr2* in macrophages after stimulation with IL4 (*n* = 4, ** *p*-value < 0.01). (**D**) Number of transmigrated E0771-LG cells in response to WT BMDMs treated with 100 µM SB332235 at the bottom chamber, recombinant CXCL1 in the receiving chamber or both (*n* = 3; **** *p*-value < 0.0001; ** *p*-value < 0.01). (**E**) Surface expression of CXCR2 determined by flow cytometry on 3B-11, Met-1, E0771-LG and WT BMDM (blue curve) compared to isotype control (light gray) and unstained control (dark gray). (**F**) Transendothelial migration of Met-1 cells in response to WT or CXCR2^−/−^ BMDM isolated from global knock-out Balb/c mice pretreated with recombinant IL4 (ANOVA and Dunnett correction, *n* = 3; * *p*-value < 0.05). (**G**) Transendothelial migration of E0771-LG cells in response to WT or *Cxcr2^fl^*^/*fl*^ BMDM with or without Cre expression isolated fromLysM-Cre-CXCR2^fl/fl^ mice (ANOVA and Dunnett correction, *n* = 4; ** *p*-value < 0.01). For all graphs, bars represent averages normalized to control +/− SD.

To investigate how IL4 modulates macrophage activity during tumor cell transendothelial migration, downstream transcriptional targets of IL4 signaling in BMDMs were examined. Using an Affimetrix dataset (GEO accession number GSE35435), the gene expression profiles of untreated BMDMs were analyzed using Ingenuity (Core) Pathway Analysis (IPA) and compared to IL4-treated BMDMs. As expected, the top networks and top biological functions altered by IL4 treatment were strongly associated with developmental and immune responses (Figure 4A). Among the top canonical signaling pathways affected by IL4 treatment, *IL-8 signaling* was identified (mice lacked IL-8 orthologs but the homologues are CXCL1, CXCL2 and CXCL5). Consequently, we investigated the expression of mRNA CXCL1, CXCL2 and CXCL5 in response to IL4 by qRT-PCR. BMDMs stimulated with IL4 highly suppressed mRNA expression of both CXCL1 (*n* = 3; *p*-value < 0.001) and CXCL2 (*n* = 3; *p*-value < 0.0001), whereas CXCL5 did not show significant differences in transcript abundance, perhaps because of the variability in response between replicates for this molecule (*n* = 3; *p*-value = 0.40) (Figure 4B).

To investigate whether IL4 stimulation of macrophages modulates transcript abundance of CXCR2 (the receptor for CXCL1 and CXCL2), CXCR2 expression was evaluated in WT BMDMs after treatment with IL4. We observed a significant five-fold increase in the CXCR2 transcript level after exposure to IL4 (*n* = 4; *p*-value < 0.05) (Figure 4C). To test the role of CXCR2 signaling in macrophages during tumor cell transendothelial migration in vitro, CXCR2 signaling was blocked during the eTEM assay. This pharmacological inhibition of CXCR2 was attained using a specific inhibitor (SB332235) which targets CXCR2 but not the closely related receptor CXCR1 [40]. SB332235 effectively inhibited AKT phosphorylation induced by treatment with CXCL1 or CXCL2 in BMDMs (Supplemental Figures S2C and S3). SB332235 strongly inhibited tumor cell transendothelial migration when added to macrophages at the bottom of the transwell system (Figure 4D). CXCR2 inhibition also suppressed the increase in tumor cell transendothelial migration in response to CXCL1 (Figure 4D). These results suggest that CXCR2 signaling is important for tumor cell transendothelial migration. To evaluate whether pharmacological inhibition of CXCR2 signaling is specific to macrophages, the level of CXCR2 expression in all cellular components of the eTEM assay was measured at the protein level by flow cytometry. All cellular components of the eTEM assay, 3B-11 endothelial cells, Met-1 and E0771 tumor cells, as well as BMDMs, expressed CXCR2 (Figure 4E), indicating that the reduction in tumor cell transendothelial migration observed with pharmacological inhibition of CXCR2 was not necessarily a result of specific inhibition of macrophages. Therefore, to unequivocally evaluate the specific contribution of CXCR2 signaling in macrophages during tumor cell transendothelial migration, we isolated BMDMs from *Cxcr2*^−/−^ mice and compared them to WT BMDMs in the eTEM assay. As observed with *IL4Rα^−^*^/*−*^ BMDMs, there was no difference in the number of transmigrated tumor cells in response to *Cxcr2*^−/−^ compared to WT BMDMs (Met-1 cells, *n* = 3, *p*-value = 0.92) (Figure 4F). Since WT macrophages stimulated with IL4 showed an increased level of *Cxcr2* transcripts, it was determined whether increased levels of CXCR2 induced by IL4 mediate the IL4-dependent increased transendothelial migration of tumor cells. To perform this experiment, BMDMs were isolated from mice with a global targeted deletion of *Cxcr2*^−/−^ carried on a BALB/c genetic background. BMDMs derived from WT and *Cxcr2*^−/−^ mice treated with IL4 were compared using the eTEM assay. IL4 pre-stimulation of *Cxcr2*^−/−^ BMDMs did not increase transmigration of tumor cells (*n* = 3, *p*-value = 0.56), in contrast to the enhanced effect with WT BMDMs (*n* = 3, *p*-value < 0.05) (Figure 4F). Similar results were observed with E0771-LG cells and *Cxcr2*-floxed BMDMs from C57BL/6 LysM-Cre mice, which showed effective deletion of genes in macrophages (Figure 4G). These data indicate that CXCR2 mediates the IL4-dependent increase in tumor cell transendothelial migration of macrophages in vitro.

### 3.5. IL4 Regulates Genes in Macrophages Required for Metastatic Seeding and Expansion

**Figure 5:**
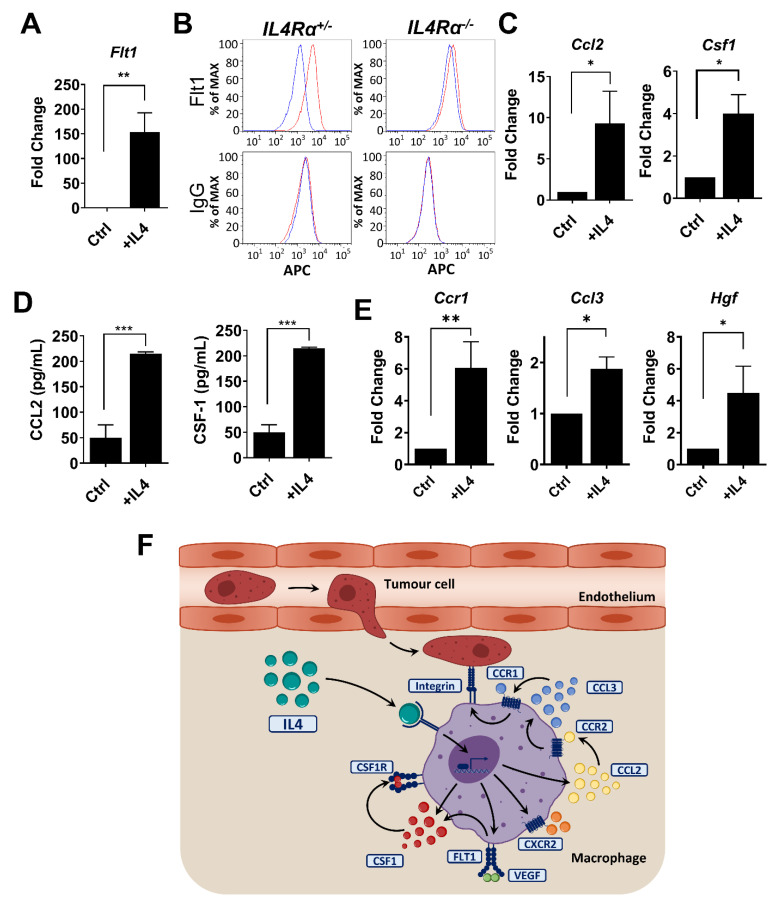
IL4 signaling controls the expression of *Flt1* and other molecules associated with metastatic progression. (**A**) mRNA expression of *Flt1* in BMDM measured by qPCR in response to 24 h stimulation with rIL4 (*n* = 4; ** *p* value < 0.01). (**B**) FLT expression on *IL4Rα*^+/−^ and *IL4Rα*^−/−^ BMDM cell surface in control conditions (blue line) and after stimulation with rIL4 (red line) measured by flow cytometry. Graph represents the mean fluorescence intensity for each condition. (**C**) mRNA expression of *Ccl2* and *Csf1* in BMDM measured by qPCR in response to 24 h stimulation with rIL4 (*n* = 4; * *p* value < 0.05). (**D**) CCL2 and CSF-1 protein expression by BMDM after 24 h of stimulation with rIL4 determined by ELISA (*n* = 3, *** *p*-value < 0.005 and ** *p*-value < 0.01). (**E**) Normalized levels of *Ccr1*, *Ccl3* and *Hgf* mRNA expression in BMDM from *Cxcr2^fl^*^/*fl*^ mice after 24 h of stimulation with rIL4, measured by qPCR (*n* = 3, ** *p*-value < 0.01 and * *p*-value < 0.05). (**F**) Proposed model for the action of IL4 effects on macrophages in the metastatic site supported by the data presented in this study and genetic analysis in previous studies.

Within the gene list related to IL-8 signaling identified by IPA in IL4-stimulated macrophages, *Flt1* was the most upregulated gene. FLT1 is important for the pro-tumoral activities of MAMs in the lung but not for their recruitment [13]. IL4 stimulation of BMDMs induces a significant increase in *Flt1* expression at the transcript level determined by qRT-PCR (*n* = 4, *p*-value < 0.01) (Figure 5A). IL4 signaling also induced FLT1 cell surface protein expression (Figure 5B). BMDMs from *IL4Rα*-null mice failed to show an increase in FLT1 on their membrane over the base line expression in response to IL4 (Figure 5B). Importantly, other genes associated with macrophage functions during metastatic progression, such as *Ccl2* and *Csf1*, were also controlled at the mRNA and protein level by IL4 signaling (Figure 5C,D) [13,18]. A further qRT-PCR assessment of genes previously shown to be important in lung experimental metastasis assays [18,41] revealed upregulation by IL4 at the transcript level of *Ccr1*, *Ccl3* and *Hgf* (Figure 5E). These data suggest that IL4 signaling can be a master regulator that controls multiple downstream effectors in MAMs that have been shown by genetic ablation or antibody inhibition to be important during tumor cell metastatic seeding (CCL2, CCR1) and growth (FLT1, CSF1) in vivo (Figure 5F).

## 4. Discussion

In the metastatic cascade, tumor cells escape into the circulation via the circulatory or lymphatic system and transit to metastatic sites where they extravasate [42]. These sites are often pre-conditioned by systemic influences of cancer, causing the creation of the so-called pre-metastatic niche [43]. These niches are often populated by myeloid cells [44]. Tumor cells can travel either singly or in clumps, but in either case, the rate-limiting steps are in their survival and subsequent establishment as metastatic lesions [5,45]. In human breast cancer, the major sites of metastasis are the bone and lung, and these are the major causes of mortality in this disease, accounting for ~90% of deaths [46]. To study these diseases, we and others have developed experimental and spontaneous mouse models of lung and bone metastases [5]. In these models of metastasis, once the tumor cells arrive, there is an immediate CCL2-mediated recruitment of CCR2-expressing classical monocytes that promote metastasis seeding and establishment [16]. In the lung, these classical monocytes differentiate via a progenitor state (MAMPC) into mature MAMs, each step promoting a different aspect of tumor cell growth into a metastatic lesion [18]. Thus, removal of these cells by blocking their recruitment, function or survival inhibits metastasis [16]. In this study, we show that IL4, classified as a Th2 cytokine, regulated the function of these cells through type 1 IL4 (IL4a)receptor signaling. This signaling induced a gene expression cascade in monocytes as they differentiate into MAMs that included the upregulation of *Flt1*, *Csf1*, *Cxcr1*, *Hgf* and *Ccr1*. IL4 is therefore a master regulator of monocytes and MAMs, causing them to promote the seeding, survival and persistent growth of metastatic cells.

During cancer evolution, it has been suggested that the tumor microenvironment needs to become immunosuppressive, so that tumors escape immune responses and thus flourish [14,47]. Evidence for this immunoregulation is partly shown by tumors that in some contexts thrived in Th2 biased (Balbc) but were rejected in Th1 mouse strains (BL6) [48,49]. In this case, rejection was mediated by iNOS-expressing macrophages in the BL6 mice, while in the Balbc strain tumor, growth was promoted by macrophage-derived arginase that is immunosuppressive. Consequently, the macrophages in these tumors from these two strains were labeled as M1 or M2 to match the Th1/2 nomenclature [48]. In tissue culture, Th1 cytokines IL12, IL18 and IFNγ and Th2 cytokines IL4, IL10 and IL13 cause macrophages to differentiate into an activated or alternatively activated state, respectively. These states are characteristic of in vivo responses to bacterial or helminth infections, respectively [50,51]. Thus, the original M1 to M2 definition was extended to encompass these activated (M1) and alternatively activated (M2) states with the consequent suggestion that M2s are tumor-promoting [52,53,54]. Subsequent research in mouse models of cancer indicated in primary tumors that IL4 is an essential cytokine responsible for macrophage differentiation to support a state that promotes macrophage-enhanced tumor cell invasion and thus tumor malignancy [21,55].

While the function of IL4 in the primary tumor has been well-studied, its role in metastases, particularly those of the lung, has not been explored. In the current study, using models of breast cancer metastasis to the lung, we indicate that IL4 regulates aspects of the differentiation of monocytes to MAMs. Consistent with this action, ablation of IL4 signaling inhibited metastatic cell colonization and persistent growth. Furthermore, IL4 stimulated the expression of several downstream genes that in previous experiments have been shown to be essential for this process (Figure 5F). This signaling includes expression of CCR1 required for MAM adhesion to extravasated tumor cells that in turn delivers a survival signal to these cells [18]. IL4 also stimulates FLT1 cell surface expression, which makes the MAMs responsive to VEGFA and CSF1, which acts in an autocrine fashion through the CSF1R and stimulates macrophage survival and polarization [13]. These signaling pathways in MAMs promote metastatic growth [13,18]. During these processes, MAMs differentiate via a precursor that is characterized by a Th2 gene expression signature and is immunosuppressive to cytotoxic T cells through superoxide formation [15]. These cells are often referred to as myeloid-derived suppressor cells. Upon their differentiation to MAMs, driven by CSF1R and FLT1 signaling, they obtain a new suppressive activity to cytotoxic T-cell killing via expression of CTLA-4 check-point inhibitor ligands [15]. In addition, IL4 induces expression of HGF, which creates an immunosuppressive environment for cytotoxic natural killer cells via c-MET signaling in tumor cells [41]. Thus, in this model of breast cancer lung metastasis, IL4 potentiates an immunosuppressive Th2 environment through its actions on monocyte differentiation to MAMs after their recruitment via CCL2 [16].

Monocytes and MAMs, in addition to creating an immunosuppressive environment, also promote the earliest steps in metastasis by enhancing metastatic cell extravasation. This action is in part due to the production of VEGFA, which increases vascular permeability [16]. The eTEM assay in our hands has been valuable to analyze this step of extravasation. This study showed that IL4 was not required for macrophage enhancement of the transendothelial migration of tumor cells in vitro but did enhance the macrophage’s ability to promote this process. Although we were unable to evaluate extravasation in vivo, our data suggest that extravasation is a limiting step and this step is promoted by MAMs [5]. IL4 upregulates CXCR2, whose genetic deletion specifically in macrophages inhibits their ability to promote this process. IL4 also downregulated the ligands CXCL1 and 2, suggesting that this removal of an autocrine response might promote paracrine signaling through CXCR2.

Importantly in the studies reported here, we also used a novel quantitative intravital imaging method to analyze interactions between tumor cells and monocytes/MAMs during the stages of intravasation in vivo. These data unequivocally show that IL4 signaling enhanced tumor cell–MAM interactions and stabilized the association between these two cell types. Previous studies indicated that this association was through the tumor cell surface expression of VCAM1 and alphaV integrin on the MAMs, whose activity was stimulated by CCR1 signaling [18]. This engagement delivers a survival signal to the tumor cells and thereby promotes metastatic cell colonization [18,56]. It also promotes monocyte/MAM retention at the metastatic site [18]. IL4 stimulates this process at least in part by upregulation of CCR1 and CCL2 expression. CCL2, in turn, upregulates CCL3 expression, which stimulates CCR1 activity [56]. These real-time in vivo experiments therefore validate the activities found in vitro [18] and indicate that IL4 is a major regulator of the process, as summarized in Figure 5F.

While the experimental metastasis methodology used in this study has its limitation because cells highly selected for colonization in the bone or lung are given as a bolus of single cells in high concentration, it does allow dissection of the process without influences of the primary tumor that can confound interpretation. With this assay, we showed reduced foci number and size in absence of IL4 signaling in macrophages. Our in vitro data suggest that IL4 signaling is relevant for extravasation and subsequent survival of tumor cells; however, the exact step requires elucidation in vivo. IL4 signaling in monocytes could have had a role during tumor cell survival in circulation, via extravasation as our eTEM suggests, or in seeding, dormancy or subsequent growth, all of which could translate into a reduced foci number. Importantly, consistent with the results obtained in the experimental metastasis model, null or conditional mutations or specific inhibitors (antibodies/small molecules) in almost all the genes described in this study, including *IL4Ra*, *Flt1*, *Ccl3*/*Ccr1*, *Vegfa* and *Csf1*/*Csf1r*, inhibited spontaneous metastasis in mouse models of breast cancer [13,18,21]. In these cases, both FLT1 and CSF1R signaling have been shown to influence metastatic growth but not seeding [13]. In models of breast cancer bone metastasis, a tissue where autochthonous tumors do not metastasize in mice, classical monocyte recruitment has also been shown to be essential for metastatic progression [27]. These monocytes differentiate into bone MAMs (BoMAMs) that upregulate IL4Ra and whose signaling promotes metastatic outgrowth as well as seeding [27]. Furthermore, IL4R-positive BoMAMs have been shown in osteolytic lesions caused by human breast cancer metastasis [27]. Altogether, IL4 signaling appears to be relevant for multiple steps of the metastatic cascade for both the bone and lung. Therefore, these data suggest that IL4R inhibition might be a therapeutic avenue to inhibit Th2 suppression of the cytotoxic immune response and enhance immunotherapy for deadly bone and lung metastatic disease.

## 5. Conclusions

The Th2 cytokine IL4 enhanced macrophage-dependent tumor cell extravasation in vitro while classical Th1 inflammatory signals inhibited this process. IL4 receptor (*IL4rα)*-null mice developed fewer and smaller lung metastases. This inhibition of the metastatic burden in *IL4rα*-deficient mice was alleviated by adoptive transfer of wild-type monocytes. Intravital imaging experiments showed that the physical interaction between tumor cells and metastasis-associated macrophages (MAMs) in the metastatic lung was reduced in *IL4rα*-deficient mice. This interaction with wild-type MAMs enhanced tumor cell survival and seeding, which was lost in the *IL4rα* mice. IL4 signaling in macrophages controlled the expression of CXCR2, which is necessary for IL4-mediated tumor cell extravasation in vitro. IL4 signaling in macrophages also transcriptionally regulated other genes causally associated with mammary cancer lung metastasis, including *Ccl2*, *Csf1*, *Ccr1*, *Hgf* and *Flt1*. These data indicate that IL4 signaling in monocytes and macrophages plays a key role during seeding and growth of breast metastasis in the lung and suggest that this pathway could be a therapeutically target to inhibit metastasis. 

## Data Availability

Not applicable.

## Supplementary Materials

The following supporting information can be downloaded at: https://www.mdpi.com/article/10.3390/cancers14174336/s1, Figure S1: In vitro extravasation assay; Figure S2: IL4rα-null mice have normal numbers of circulating classical and non-classical monocytes. Figure S3: WB original image. Video S1: Intravital imaging of a tumor cell in the intravascular space soon after arrival at the lung and its interaction with a CFP+ monocyte in a WT mouse. Video S2: Intravital imaging of a tumor cell in the extravascular space, 24 h after and its interaction with a CFP+ macrophage in the lung of a WT mouse. Video S3: Intravital imaging of a tumor cell in the extravascular space, 24 h after and its interaction with a CFP+ macrophage in the lung of a Il4R*α*^−/−^ mouse.
