# Supplementary material for: Interleukin 4 Controls the Pro-Tumoral Role of Macrophages in Mammary Cancer Pulmonary Metastasis in Mice
Source: Cancers (Basel). 2022 Sep 5;14(17):4336. doi: 10.3390/cancers14174336 (PMC9454655; doi:10.3390/cancers14174336)
Supplement: Supplementary file 1 [file cancers-14-04336-s001.zip › Supplementary Figures.pdf]

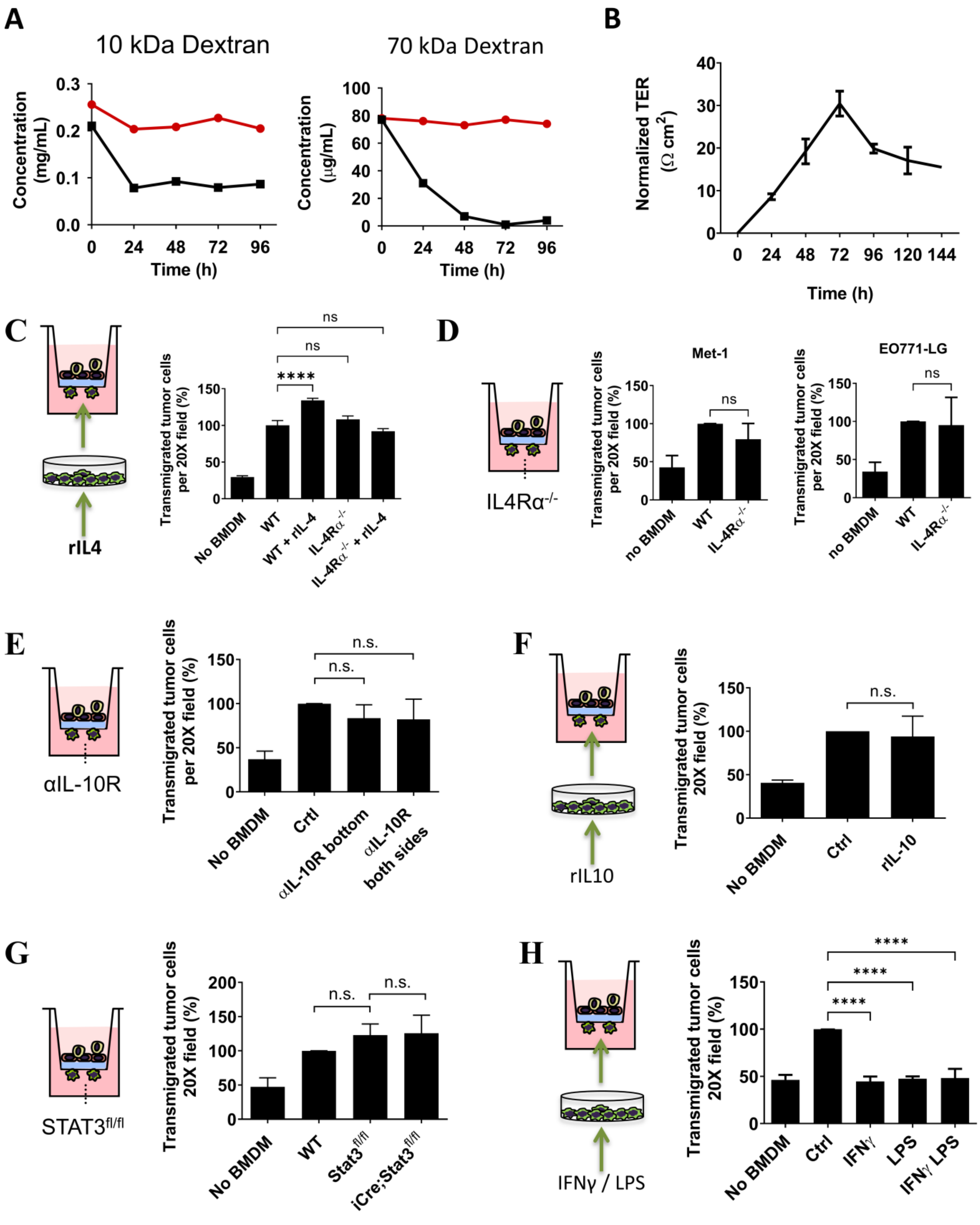

Figure S1. In vitro extravasation assay.

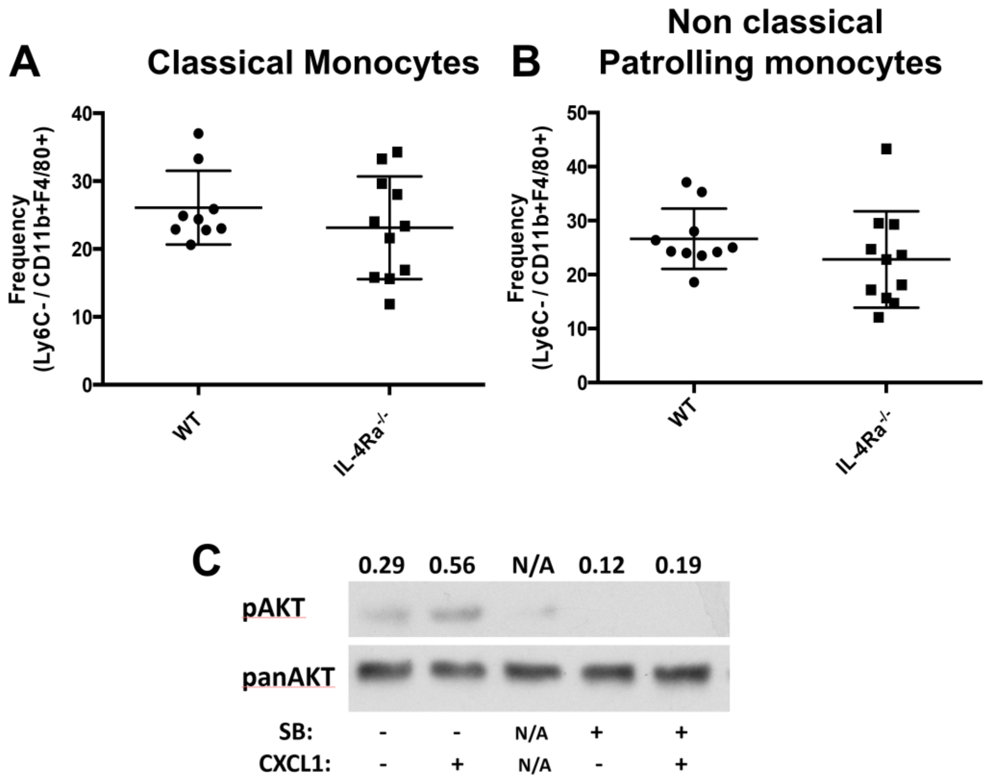

Figure S2. IL4rα-null mice have normal numbers of circulating classical and non-classical monocytes**.**

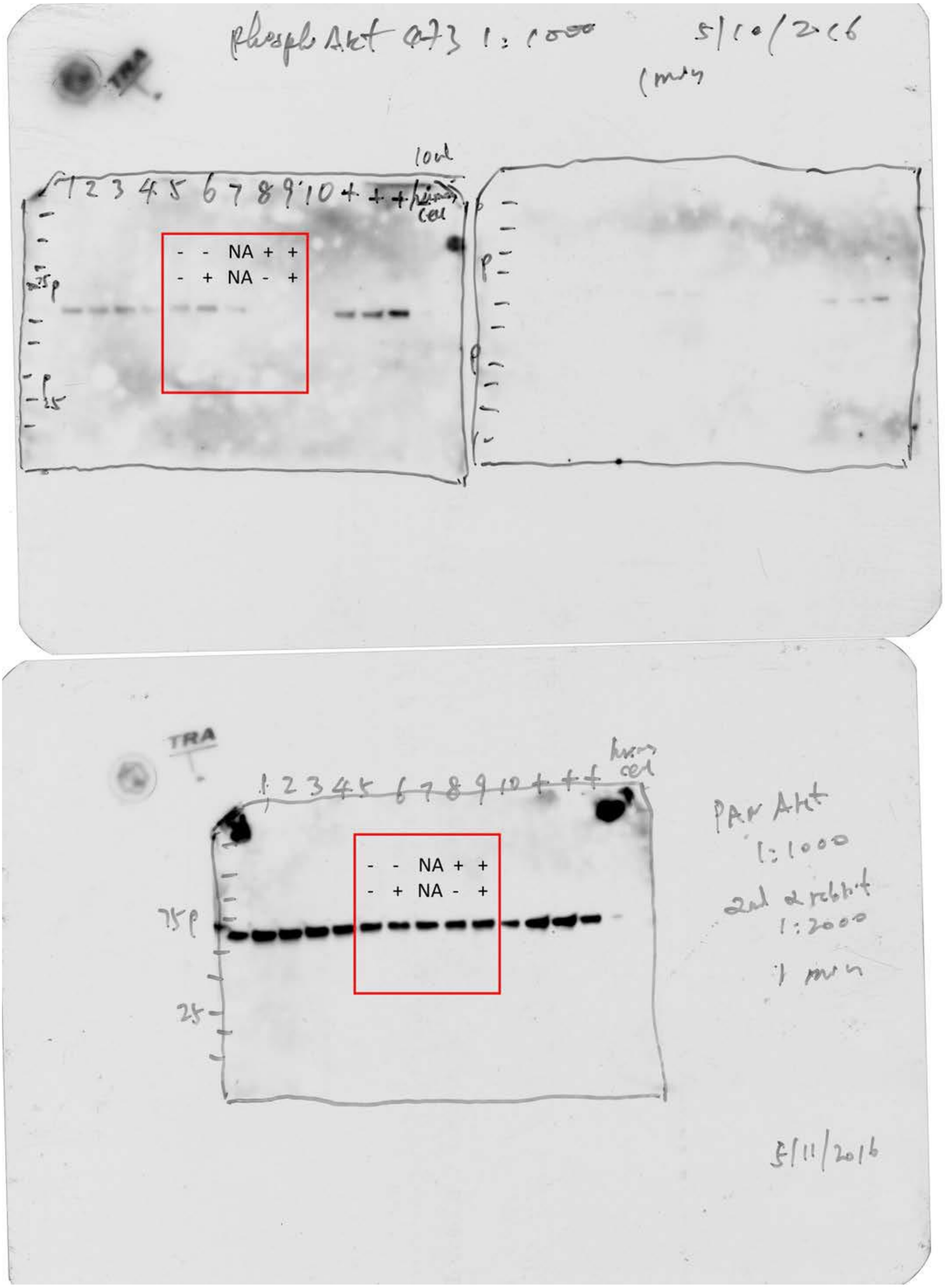

Figure S3. WB original image.
